# Serum IgG Anti-*Toxoplasma gondii* Antibody Concentrations Do Not Correlate Nested PCR Results in Blood Donors

**DOI:** 10.3389/fcimb.2019.00461

**Published:** 2020-01-14

**Authors:** Fabiana Nakashima, Valquíria Sousa Pardo, Marcos Paulo Miola, Fernando Henrique Antunes Murata, Natalia Paduan, Stefani Miqueline Longo, Cinara Cássia Brandão de Mattos, Vera Lucia Pereira-Chioccola, Octávio Ricci, Luiz Carlos de Mattos

**Affiliations:** ^1^Immunogenetics Laboratory, Molecular Biology Department, Faculdade de Medicina de São José do Rio Preto, São Paulo, Brazil; ^2^Blood Bank São José do Rio Preto, Fundação Faculdade Regional de Medicina, São Paulo, Brazil; ^3^FAMERP Toxoplasma Research Group, São Paulo, Brazil; ^4^Parasite Molecular Biology Laboratory, Instituto Adolfo Lutz, São Paulo, Brazil

**Keywords:** *Toxoplasma gondii*, serology, molecular diagnosis, Nested PCR, serology assay, transfusion, blood donation and transfusion, blood donnors

## Abstract

**Background:** *Toxoplasma gondii* infects millions of individuals worldwide. This protozoan is food and water-borne transmitted but blood transfusion and organ transplantation constitute alternative forms for transmission. However, the influence of IgG anti-*T. gondii* antibodies in molecular analysis carried out in peripheral blood still remain unclear. This study aimed to investigate the serum IgG anti-*T. gondii* antibody concentrations correlate Nested PCR results in blood donors.

**Methods:** 750 blood donors were enrolled. IgM and IgG anti-*T. gondii* antibodies were assessed by ELISA (DiaSorin, Italy). Nested PCR was performed with primers JW62/JW63 (288 bp) and B22/B23 (115 bp) of the *T. gondii B1* gene. The mean values of IgG concentration were compared for PCR positive and PCR Negative blood donors using the *t*-test or Mann-Whitney according to the normal distribution (*p*-value ≤ 0.05).

**Results:** 361 (48.1%) blood donors presented positive serology as follow: IgM^+^/IgG^−^: 5 (0.6%); IgM^+^/IgG^+^: 21 (2.8%); IgM^−^/IgG^+^: 335 (44.7%) and 389 (51.9%), negative serology. From 353 blood donors with positive serology tested, the Nested PCR was positive in 38 (10.8%) and negative in 315 (89.2%). There were no differences statistically significant between the mean values of serum IgG anti-*T. gondii* antibody concentrations and the Nested PCR results.

**Conclusions:** In conclusion, our data show that variations in the serum IgG anti-*T. gondii* antibody concentrations do not correlate *T. gondii* parasitemia detected by Nested PCR in chronically infected healthy blood donors.

## Background

The infection by *Toxoplasma gondii* is frequent around the world and its prevalence range from <30% to more than 60% (Pappas et al., 2009; Dubey et al., 2012; Wallon and Peyron, 2018; Greigert et al., 2019). Different clinical forms of toxoplasmosis resulting from the infection by this Apicomplexan parasite arises and drawn attention especially for pregnant women, newborns and other immunosuppressed patients (Robert-Gangneux and Dardé, 2012; Neu et al., 2015; Rostami et al., 2019; Vidal, 2019).

*T. gondii* infection is a subject of social, epidemiological, clinical and scientific interest in Brazil. The rates of seroprevalence and the great genomic diversity of this parasite are high around the country (Dubey et al., 2012). Among the different ways to transmit *T. gondii* infection, the transfusion of blood products has been less explored. Two studies carried out in the past reported transmission of *T. gondii* by transfusion of leucocytes and platelets but the authors reached their conclusions after exclude other potential ways by which this parasite could be transmitted (Siegel et al., 1971; Nelson et al., 1989). Even so, this matter still represents a challenge for contemporaneous transfusion medicine (Foroutan et al., 2018; La Hoz et al., 2019).

The diagnosis of infection by *T. gondii* is essentially serological but there are number of published papers demonstrating that the high sensitivity of molecular methods can offer more accurate results on the investigation of infection by this parasite (Mattos et al., 2011; Brenier-Pinchart et al., 2015; Robert-Gangneux et al., 2015; Camilo et al., 2017; Murata et al., 2017; Roux et al., 2018; Greigert et al., 2019; Lévêque et al., 2019; Pleyer et al., 2019). The combination of serology and molecular methods has been used to improve the diagnosis of infection by *T. gondii*. One of them reported that IgG anti-*T. gondii* antibody low avidity correlates positive PCR in pregnant women (Yamada et al., 2011; Murata et al., 2016, 2017; Olariu et al., 2019). The other one also showed that IgG anti-*T. gondii* antibody low avidity correlate positive PCR among patients with ocular toxoplasmosis (Costa-Silva et al., 2008; Mattos et al., 2011; Tsirouki et al., 2018; Cortés et al., 2019; Greigert et al., 2019; Rahimi Esboei et al., 2019). However, correlations between serum anti-*T. gondii* antibody concentrations and molecular diagnosis of *T. gondii* infection among blood donors are scarce in the literature.

Evaluation of the serum IgG anti-*T. gondii* antibody concentrations could contribute to the understanding of the importance of these antibodies as risk biomarkers for transfusional purposes in respect to *T. gondii* transfusional transmission. The aim of this study is to test the hypothesis that low serum concentrations of IgG anti-*T. gondii* antibodies correlate *T. gondii* parasitemia.

## Methods

### Ethics Considerations

This study was approved by Research Ethics Committee from Faculdade de Medicina de São José do Rio Preto (case 006/2011). All blood donors received information about the objectives of the study and gave their informed consent.

### Selection of Blood Donors

We selected a total of 750 blood donors from both genders able to donate at Regional Blood Center from São José do Rio Preto. All of them were seronegative for other infectious diseases as required by Brazilian policy for blood donation—B and C hepatitis, HIV, Chagas, syphilis, HTLV I/II (Ministério da Saúde, 2011).

### Blood Sampling

Two blood samples were obtained from each blood donor by venipuncture from peripheral blood. One of them was collected with EDTA as anticoagulant and used to DNA extraction. The other one was collected without anticoagulant and stored at −20°C until used for detection of IgM and IgG anti-*T. gondii* antibodies.

### Serology Assays for IgM and IgG Anti-*T. gondii* Antibodies

Serological tests for specific IgM and IgG antibodies anti-*T. gondii* were carried out by a commercial immunoenzymatic assay kit (DiaSorin, Italy). IgG anti-*T. gondii* antibody concentrations were defined according to the calibrators representing the cut-off values. All the manufacturer's instructions were precisely followed.

### DNA Extraction

Genomic DNA of buffy coat from 5 mL of blood samples collected with EDTA was extracted using PureLink Genomic DNA Kits (Invitrogen, Carlsbad, CA), as previously described (Mattos et al., 2011).

### PCR Nested Molecular Analysis

Nested PCR was performed using the *B1* gene (accession numbers: *B1* gene *T. gondii* = GenBank: KR559682.1) of *T. gondii* genomic DNA was carried out according to the protocol published by Okay and colleagues (Okay et al., 2009).

The first PCR reaction used the set of primers JW62 (Anti-sense: 5′-TTCTCGCCTCATTTCTGGGTCTAC-3′) and JW63 (Sense: 5′-GCACCTTTCGGACCTCAACAACCG-3′) to amplify a fragment of 288 base pairs. The composition of the mix for each reaction with 25 μL of final volume was: 0.2 μL of each primer, 100 ng of genomic DNA and 1× of Go Taq Green Master Mix (Promega, USA). The conditions of amplification were: 1× initial denaturation at 95°C: 5 min, 40× (denaturation at 95°C: 45 s, annealing at 55°C: 45 s, extension at 72°C: 45 s), 1× final extension at 72°C: 5 min, final at 4°C: 30 min. The amplified fragments were electrophoresed in 2% agarose gel stained with ethidium bromide under UV light.

The second PCR reaction used the set of primers B22 (Sense: 5′-AACGGGCGAGTAGCACCTGAGGAGA-3′) and B23 (Anti-sense: 5′-TGGGTCTACGTCGATGGCATGACAACT-3′) to amplify a fragment of 115 base pairs. The composition of the mix for each reaction with 25 μL of final volume was: 1.2 μM of each primer, 0.5 μL of pre-amplified DNA and 1× of Go Taq Green Master Mix (Promega, USA). The conditions of amplification were: 1× initial denaturation at 95°C: 5 min, 45× (denaturation at 95°C: 45 s, annealing at 55°C: 45 s, extension at 72°C: 45 s), 1× final extension at 72°C: 5 min, final at 4°C: 30 min. The amplified fragments were electrophoresed in 2% agarose gel stained with ethidium bromide under UV light.

### Statistical Analysis

The mean values of IgG concentration were compared for blood donors with positive and negative PCR using the *t*-test or Mann-Whitney according to the normal distribution. The level of significance was set at 5% (*p*-value ≤ 0.05). The GraphPad Instat® (GraphPad Software Inc., USA) computer program version 3.06 was used for all analyses.

## Results

**Figure 1:**
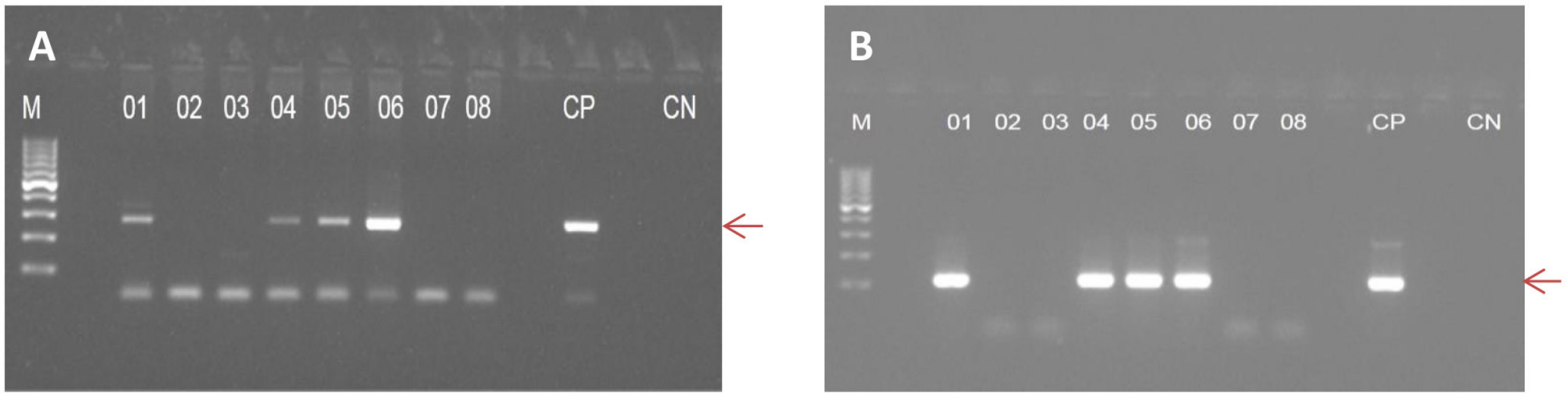
Electrophoretic profile of fragments of *B1* gene from *T. gondii* genomic DNA extracted from peripheral blood of blood donors, amplified by Nested PCR. In **(A)**, the first amplification is showing a fragment of 288 base pairs; in **(B)**, the second amplification is showing a fragment of 115 base pairs. M, molecular marker; PC, Positive Control; CN, Negative Control.

The serology results and their interpretation are shown in Table 1. From the overall blood donors able to donate (*n* = 750), 244 were female (mean age: 32.7 ± 10.7 years), and 506 were male (mean age: 34.7 ± 11.6) (*p* = 0.097). The IgM^+^/IgG^+^ serology was more frequent in males than in females (*p* = 0.0308). We performed the Nested PCR in 353 blood donors carrying serum IgG antibodies (IgM^+^/IgG^+^ and IgM^−^/IgG^+^). Figure 1 shows the amplified fragment from the genomic DNA of *T. gondii* extracted from peripheral blood carrying 288 and 115 bp, respectively.

**Table 1:** Serology results and the interpretation of the serological profiles and Nested PCR for blood donors.

**Serology**			Interpretation^*^	**Male**		**Female**		**OR**	**CI 95%**	p^**^
	***n***	**%**		***n***	**%**	***n***	**%**			
IgM^+^/IgG^−^	5	0.6	Recent infection	3	0.6	2	0.8	0.721	0.119–4.349	0.662
IgM^+^/IgG^+^	21	2.8	Recent infection	19	3.7	2	0.8	4.721	1.090–20.440	0.030
IgM^−^/IgG^+^	335	44.7	Chronic infection	233	46.0	102	41.8	1.188	0.872–1.618	0.308
IgM^−^/IgG^−^	389	51.9	Non-immunized	251	49.7	138	56.6	0.756	0.556–1.028	0.086
Total	750	100.0		506		244				

* According to Montoya (2002).

** *Calculated by exact Fisher's test*.

There were no differences statistically significant between the mean serum IgG anti-*T. gondii* antibody concentrations and positive or negative Nested PCR results even when the comparisons were made by gender. Table 2 shows the data from male and female blood donors with positive and negative Nested PCR and serum IgG anti-*T. gondii* antibody concentrations.

**Table 2:** Mean age, median, range, normal distribution, IgG anti-*T. gondii* antibodies concentrations according to Nested PCR Positive and Negative in male and female healthy blood donors.

**Values**	**Nested PCR (*****N*** **= 353)**			
	**Positive (*****n*** **= 38)**		**Negative (*****n*** **= 315)**	
	**Male (*n* = 31)**	**Female (*n* = 7)**	**Male (*n* = 221)**	**Female (*n* = 94)**
Mean age ± SD	37.7 ± 11.2	38.7 ± 11.9	37.9 ± 10.9	35.0 ± 10.5
Median	38	33	37	34
Range	19–60	23–52	18–65	18–59
25th Percentile (range)	28 (19–37)	23 (23–32)	28 (18–36)	27 (18–33)
75th Percentile (range)	47 (39–60)	51 (50–52)	46 (38–65)	43 (34–59)
Normal distribution	Yes	Yes	Yes	No
IgG^+^				
IgG ± SD (UI/mL)	196.0 ± 35.2	165.6 ± 62.7	204.0 ± 33.2	196.6 ± 39.5
Median	205.6	186.8	208.3	205.9
Range	66.1–232.1	36.1–222.8	21.4–257.3	55.2–244.7
25th Percentile (range)	171.9 (66.1–202.1)	145.1 (36.1–168.9)	180.1 (21.4–208.2)	169.6 (55.2–205.8)
75th Percentile (range)	219.5 (206.0–232.1)	212.5 (186.9–222.8)	227.9 (208.4–257.3)	223.6 (205.9–244.7)
Normal distribution	No	Yes	No	No

*OBS: Male-PCR+ vs. Male-PCR–: p = 0.2252 (Mann–Whitney U′ = 3,887.0); Female-PCR+ vs. Female-PCR–: p = 0.0960 (Mann–Whitney U′ = 454.00); Male-PCR+ vs. Female-PCR+: p = 0.1065 (Mann–Whitney U′ = 152.00); Male-PCR– vs. Female-PCR–: p = 0.2486 (Mann–Whitney U′ = 11,241.00)*.

## Discussion

The aim of this study was to test the hypothesis that serum concentrations of IgG anti-*T. gondii* antibodies correlate *T. gondii* parasitemia in healthy blood donors. As the screening for anti-*T. gondii* antibodies is not compulsory for blood donors in Brazil (Ministério da Saúde, 2011) we performed serological tests to detect IgM and IgG anti-*T. gondii* antibodies as well as Nested PCR targeting *B1* gene from *T. gondii* to detect parasitemia.

Serum IgM and IgG anti-*T. gondii* antibodies have been investigated in blood donors aiming to determine the prevalence of infection in different countries as reviewed by Foroutan-Rad and colleagues (Foroutan-Rad et al., 2016; Foroutan et al., 2018) as well as to estimate the risk of transfusional transmission of this parasite (Siransy et al., 2016; Ferreira et al., 2017; Botein et al., 2019; El-Tantawy et al., 2019). However, correlations between serum IgG anti-*T. gondii* antibody concentrations and the parasitemia determined by molecular methods have not been explored in healthy blood donors. A correlation between high serum IgG anti-*T. gondii* concentrations and negative PCR could be explored as an indicator for low risk of transfusional transmission of this parasite by blood products.

In this study, we observed that none of the IgM^+^/IgG^−^ blood donors were positive for Nested PCR. Only one of the IgM^+^/IgG^+^ presented positive Nested PCR. Moreover, male and female blood donors with positive Nested PCR presented the mean values of serum IgG anti-*T. gondii* antibody concentrations lower in comparison to their counterpart with negative Nested PCR. However, the differences were not statistically significant. Therefore, low or high serum IgG anti-*T. gondii* antibody concentrations do not correlate the result of molecular analysis by Nested PCR aiming to detect genomic DNA from *T. gondii* in peripheral blood from healthy blood donors.

Molecular methods, such as conventional PCR, Nested PCR, and real-time PCR have been used either in isolation or in association, to detect *T. gondii* parasitemia in acute and chronically infected individuals since they show high sensitivity (Brenier-Pinchart et al., 2015; Dard et al., 2016; Camilo et al., 2017; Roux et al., 2018; Botein et al., 2019; Greigert et al., 2019). In this study, we used the Nested PCR to target the *B1* gene which is one of the most used tests in the literature for detecting *T. gondii* parasitemia (Okay et al., 2009; Mattos et al., 2011; Teixeira et al., 2013; Roux et al., 2018). Moreover, it has been demonstrated that the *B1* gene might be targeted for molecular detection of *T. gondii* parasitemia in Brazilian samples, especially when the investigation is limited to one gene (Okay et al., 2009; Teixeira et al., 2013).

It would be desirable to obtain *T. gondii* isolates from the blood donor or the donate samples (blood bags). However, due to the short length of parasitemia, which is apparently restricted to the acute phase of infection, is difficult to obtain viable parasites from blood samples. Maybe, an alternative would be to isolate parasite's mRNA from the donated blood but this procedure could interfere in the routine process in blood banks and contaminate the blood bags. Due to these difficulties, the studies that explored molecular approaches to detect the infection by this parasite among blood donors carry some limitations. Molecular methods aiming to detect *T. gondii* genomic DNA in the peripheral blood, such as conventional and Nested PCR are unable to distinguish live or dead parasites as well as residual DNA. They can only give a measure of the risk of transmission as well as overestimate the presence of the parasite in the peripheral blood (Rousseau et al., 2018).

The data presented here are supported by other reports. Three Iranian studies detected *T. gondii* infection only in blood donors carrying IgM anti-*T. gondii* antibodies by real-time PCR (Mahmoudvand et al., 2015) and Nested PCR (Sadooghian et al., 2017; Saki et al., 2019). In fact, there is a strong correlation between the IgM anti-*T. gondii* antibodies and parasitemia. However, parasitemia cannot be discharged in immunocompetent individuals (potential blood donors) carrying circulating IgG anti-*T. gondii* antibodies (Mattos et al., 2011; Park, 2012), especially when these antibodies present low avidity (Mattos et al., 2011; Yamada et al., 2011; Saki et al., 2019). All these studies did not correlate the serum concentration of anti-*T. gondii* antibodies to the PCR results.

The role of the host's immune response is crucial to protect chronically infected individuals (Coombes and Hunter, 2015). On the one hand, the cellular immune response led by macrophages, T CD8 lymphocytes, Natural Killer cells and cytokines as Interferon gama (IFN-ɤ) protects against the intracellular forms of the parasite. On the other hand, humoral immune response, which is thought to play a minor role in the immune protection of the host, seems to be effective against *T. gondii* extracellular forms, such as tachyzoites (Cohen and Denkers, 2015). The anamnestic immune response against *T. gondii* is characterized by the expression of IgG antibodies with high avidity and this class of immunoglobulins is effective at least in three immune events: opsonization and phagocytosis, Complement activation and Antibody-Dependent Cytotoxicity (ADCC) by Natural Killer cells and other white blood cells (Pleass and Woof, 2001; Filisetti and Candolfi, 2004; Ortiz-Alegría et al., 2010).

Erbe et al. (1991) demonstrated that human myeloid and lymphoid cells to kill *T. gondii* tachyzoites. These authors concluded that opsonization allows the binding of Fc IgG portion to Fc receptors (FcɤR) on phagocytic cells and significantly enhances the killing of tachyzoites coated by IgG anti-*T. gondii* antibodies. Additionally, Costa-Silva et al. (2012) reported that high levels of IgG from chronically infected mice decreases *T. gondii* RH strain parasitemia in comparison to those from naive mice. Exploring an experimental model, Seeber (2000) demonstrated the lytic activity mediated by Complement against *T. gondii* tachyzoites (Seeber, 2000). Other experimental study demonstrated the ability of IgG anti-excreted-secreted antigens from *T. gondii* to agglutinate tachyzoites and kill them by Complement lysis in a mouse model (Costa-Silva et al., 2008). Pleass and Woof (2001) reported that NK cells activated by IFN-γ display Fc receptor for Fc IgG portion which binds IgG anti-*T. gondii* antibodies and kills tachyzoites through ADCC (Pleass and Woof, 2001). All these observations suggest that IgG anti-*T. gondii* antibodies are effective and promote the clearance of parasitemia in chronically infected healthy blood donors.

Despite the limitations of this study which evaluated only the *B1* gene and did not determine the IgG avidity, our data confirm the potential parasitemia in blood donors with circulating IgG anti-*T. gondii* antibodies, and demonstrate that the mean values of serum concentration of these antibodies do not correlate the results of Nested PCR. Also, it supports the view that blood products collected from chronically infected blood donors constitute a risk for transfusional transmission of *T. gondii*. In conclusion, our data show that variations in the serum IgG anti-*T. gondii* antibody concentrations do not correlate *T. gondii* parasitemia detected by Nested PCR in chronically infected healthy blood donors. Therefore, the use of serum IgG anti-*T. gondii* antibody concentrations to estimate the risk of transfusional transmission of this parasite does not constitute a potential biomarker for transfusional purposes.

## Data Availability Statement

All datasets generated for this study are included in the article/supplementary material.

## Ethics Statement

The studies involving human participants were reviewed and approved by Research Ethics Committee from Faculdade de Medicina de São José do Rio Preto (case 006/2011). All blood donors received information about the objectives of the study and gave their informed consent. The patients/participants provided their written informed consent to participate in this study.

## Author Contributions

FN, CB, VP-C, and LM designed the study and wrote the manuscript. FN and FM performed the molecular tests. OR selected the blood donors. VP, NP, MM, and SL collected the blood samples from blood donors and performed the serology tests.

### Conflict of Interest

The authors declare that the research was conducted in the absence of any commercial or financial relationships that could be construed as a potential conflict of interest.
